# Transjugular liver biopsy: enlarge the indications for liver biopsy with reliable diagnostic quality

**DOI:** 10.1186/s12876-023-02917-x

**Published:** 2023-08-14

**Authors:** Miao-Yang Chen, An-Yin Yang, Yi-Fan Hu, Yong-Feng Yang, Qing-Fang Xiong, Yan-Dan Zhong, Du-Xian Liu

**Affiliations:** 1 Department of liver diseases, The Second Hospital of Nanjing, Nanjing University of Chinese Medicine, No.1 Zhongfu Road, Gulou District, Nanjing, 210003 China; 2 Department of pathology, The Second Hospital of Nanjing, Nanjing University of Chinese Medicine, No.1 Zhongfu Road, Gulou District, Nanjing, 210003 China

**Keywords:** Transjugular liver biopsy, Complication, Diagnostic efficiency, Propensity score matching

## Abstract

**Background:**

Complications and diagnostic efficiency for liver biopsy are main concerns for clinicians. This study aimed to assess the safety and efficacy of transjugular liver biopsy (TJLB) compared with percutaneous liver biopsy (PLB) when patients had equal level of liver function and number of passes, using propensity score matching (PSM).

**Methods:**

The clinical and pathological data of patients who received TJLB or PLB between January 2012 and October 2022 were collected. Matching factors included age, gender, cirrhosis, portal hypertension, liver function, creatinine, number of passes, hemodialysis, history of anti-coagulation and anti-platelet, and comorbidities. Coagulation indexes were not considered as matching factors due to different indications of the two techniques.

**Results:**

2711 PLBs and 30 TJLBs were evaluated. By PSM, 75 patients (50 PLBs, 25 TJLBs) were matched. The complication rates for TJLB and PLB were 4.0% (1/25) and 10.0% (5/50) (P > 0.05). Two PLBs had hepatic hemorrhage, one of which required only close monitoring (Grade 1) and the other needed hemostasis and rehydration therapy (Grade 2). The other 3 cases presented with mild abdominal pain (Grade 1). And only one TJLB presented with mild pain. The median number of complete portal tracts were 6.0 and 10.0 for TJLBs and PLBs (P < 0.05). Moreover, the median length of sample for TJLBs and PLBs were 10.0 and 16.5 mm (P < 0.05). The diagnostic efficiency of hepatopathy of unknown etiology of TJLB versus PLB groups before and after matching were 96.4% vs. 94.1% and 95.7% vs. 93.2%, respectively (P > 0.05).

**Conclusion:**

TJLB is an effective invasive diagnostic procedure that expands indications for liver biopsy with reliable diagnostic quality.

## Introduction

Liver biopsy (LB) is an essential tool for diagnosing and staging liver diseases. Currently, percutaneous liver biopsy (PLB) is the most common method for obtaining liver sample in clinical practice. There are several conditions in which the risk of adverse events can be greatly increased with PLB, including platelets count < 50 × 10^9^/L, international normalized ratio (INR) ≥1.5, massive ascites, or obesity [1]. In these situations, transjugular liver biopsy (TJLB) might be a safer and preferable method, but it is not meant to be a superior one. Furthermore, TJLB can simultaneously conduct measurement of hepatic venous pressure gradients (HVPG), even if there are no contraindications as described above.

Kalambokis et al. showed that the rates of minor and major complications for TJLB were 6.5% and 0.5%, respectively [2]. Compared to percutaneous liver biopsy, pain is less commonly reported with transjugular liver biopsy, and postoperative bleeding risk is comparable with both biopsy techniques [3]. Nevertheless, these studies did not match factors that related to quality of sample, liver function, and risk of complications of the patients and compare diagnostic quality of the two procedures.

In this retrospective study, we investigated the safety and efficacy of TJLB and PLB when patients have equal levels of liver function, number of passes, and other baseline characteristics (creatinine, presence or absence of anticoagulation, antiplatelet, and other comorbidities et al.), by propensity score matching (PSM).

## Materials and methods

### Patients selection

Between January 2012 and October 2022, we retrospectively collected clinico-pathological data of 2982 patients undergoing liver biopsies at the Second Hospital of Nanjing. In this retrospective study, the data were anonymous, and the requirement for informed consent was waived by Medical Ethics Committee of the Second Hospital of Nanjing. The study was approved by the examination of the Second Hospital of Nanjing (No. 2022-LY-kt101). The study protocol conformed to the ethical guidelines of the Declaration of Helsinki.

### Enrollment criteria

#### Inclusion criteria

Patients who fulfilled one of the following conditions [4, 5] were given TJLB: (1) platelet count (PLT) < 50 × 10^9^/L, (2) INR ≥ 1.5, (3) prothrombin time activity (PTA) < 60%, (4) massive ascites, (5) measurement of the hepatic venous pressure gradient was required.

#### Exclusion criteria

Patients who were biopsied for focal liver lesions and lacked data were excluded.

### Collection of clinical data

Age, gender, routine blood test, coagulation parameter, liver function test, creatinine, etiology, presence or absence of cirrhosis, portal hypertension (PH), ascites, anti-coagulation, anti-platelet, hemodialysis, and other comorbidities, number of complete portal tracts (CPTs), length of sample, number of passes and complications were collected.

### Definition of portal hypertension

One of the following conditions indicates portal hypertension [6]: (1) ascites, (2) spleen size ≥ 13 cm in the largest axis, (3) gastroesophageal, or ectopic varices or portal hypertensive bleeding, (4) portal-systemic shunts, (5) HVPG > 5 mmHg [7].

### Definition of Hepatic hemorrhage

One of the following conditions indicates bleeding events: (1) persistently decreasing postoperative blood pressure requiring rehydration, transfusions, or even surgical hemostasis, (2) a fall in hemoglobin > 2 g/dl within 6 days postoperatively that cannot be explained by other causes [8], (3) postoperative ultrasound or CT indicated abdominal hemorrhage or intrahepatic hematoma in the liver, (4) bleeding at the local site.

### Grading of complication

We graded complications according to Common Terminology Criteria for Adverse Events (CTCAE, version 5.0). Hepatic hemorrhage was graded into 5 grades: Grade 1, bleeding required only close monitoring without intervention; Grade 2, bleeding was intervention indicated; Grade 3, bleeding required transfusions or invasive intervention; Grade 4, bleeding needed intervention with surgery; Grade 5, death. Abdominal pain was graded into 3 grades, including mild pain, moderate pain (limiting instrumental activities of daily living), and severe pain (limiting self-care activities of daily living). Other complication included fever and gastrointestinal stress.

### Definition of diagnostic quality

Diagnostic quality means that the proportion of patients with a definite cause after liver biopsy, excluding those who had liver biopsy for definite viral hepatitis staging and grading.

### Matching factors

The covariates included age, gender, cirrhosis, PH, total bilirubin (TB), albumin (Alb), alanine aminotransferase (ALT), aspartate aminotransferase (AST), alkaline phosphatase (ALP), γ-glutamine transpeptidase (γ-GT), creatinine, history of anti-coagulation, anti-platelet, hemodialysis, other comorbidities. As ascites is a manifestation of portal hypertension, which has been matched. So, we did not match ascites. Moreover, coagulation indexes were not taken as matching factors due to different indications of the two techniques.

### Liver biopsy

Before the procedure, all patients completed routine laboratory and abdominal radiographic examinations (ultrasound or computed tomography). Informed patients and families of the purpose and risks of liver biopsy, and informed consent was signed by each patient.

For the procedure, patients were placed in the left-lateral (PLB) or supine (TJLB) position. All procedures were performed after disinfection and local anesthesia. PLB was performed using a 16 G needle under ultrasound-guided. TJLB entailed access to the right internal jugular vein (IJV) using an 18G needle. Then, a super-smooth guidewire was introduced through the needle, and a 9 F vascular sheath in the LABS-100 (Cook, USA) kit was inserted into the right internal jugular vein over the wire. A 5 MPA was placed into the IJV through the sheath. Injecting the contrast agent to show the patency of hepatic veins after successful intubation into the hepatic vein with digital subtraction angiography. Partial patients measured HVPG. Next, a 7 F cutting biopsy device was replaced for sampling after determining the puncture location. Electrocardiogram monitoring was carried out throughout the process.

The liver tissue was fixed in a 4% formaldehyde solution and sent to the pathology department. Asked patients to stay in bed for 24 h after the operation, and the postoperative pain, bleeding, and other complications were closely observed. Electrocardiogram monitoring was performed for 24 h.

### Statistical analyses

Data was processed by SPSS 25.0 and R software 4.2.1. To eliminate potential bias due to lack of equal distribution between the two groups, propensity scores were calculated using a logistic regression model. The nearest neighbor matching with the ratio of 1:2 was adopted, and the caliper was 0.05. Normally distributed data was expressed as the mean±standard deviation and compared by independent sample t-test. Skewed distribution data was expressed as median (IQR) and compared by non-parametric tests. Categorical data and ranked data were expressed as percentages (%) and compared by chi-square test, fisher’s precision probability test, or Mann-Whitney U test. A difference of P < 0.05 was considered statistically significant.

## Results

### Patient demographics

**Fig. 1:**
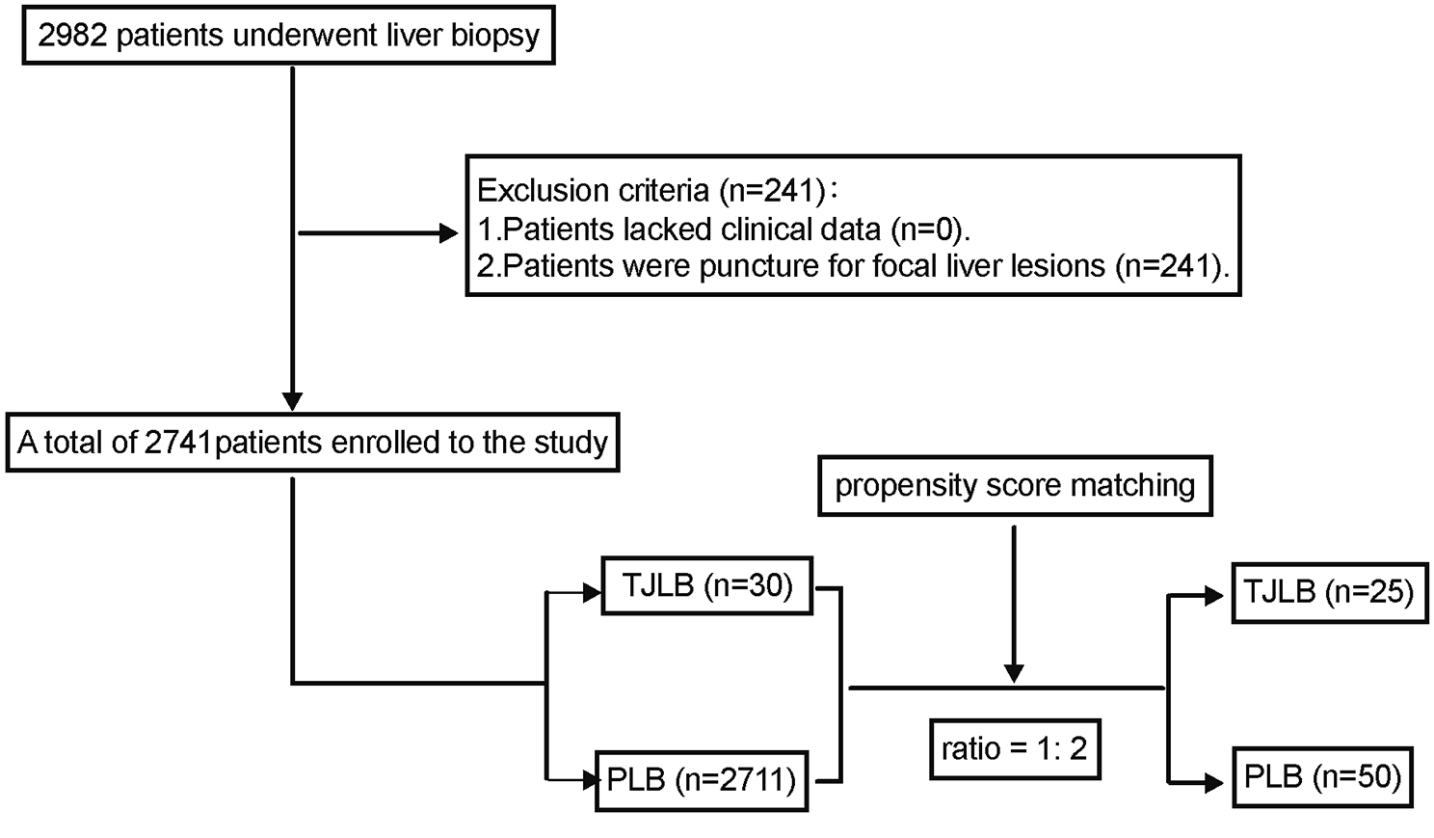
Flow diagram for enrolling study population

A total of 2982 patients underwent liver biopsy, 241 were excluded due to biopsy for focal liver lesions, and 2741 were included in the study. A total of 2711 PLBs and 30 TJLBs (Fig. 1). In TJLBs, nine patients (30.0%) had platelet counts of less than 50 × 10^9^/L, and the lowest was 13 × 10^9^/L. Two patients (6.7%) had an INR greater than 1.5, with a maximum value of 1.53. PTA was < 60% in eight patients (26.7%), and the lowest was 47.1%. Thirteen patients (43.3%) had massive ascites. Twenty-three patients (76.7%) also accepted measurement of the hepatic venous pressure gradient, with a median HVPG value of 22 mmHg.

### Clinical characteristics of patients before propensity score matching

Table 1 showed the baseline characteristics of the patients. In contrast to PLBs, TJLBs had higher percentage of age (> 50), cirrhosis (26.7% vs. 7.5%), portal hypertension (96.7% vs. 10.9%), and ascites (73.3% vs. 4.6%), lower red blood cells (RBC), hemoglobin (Hb), and ALT, and higher level of PT, INR, and TB (P<0.05). There was no significant difference between the two groups among levels of AST, ALP, and γ-GT. Complication rates of TJLB and PLB were 3.3% (1/30) and 5.6% (152/2711), with no statistical difference. In PLBs, there were 35 cases of hepatic hemorrhage, 21 of which needed only close monitoring without intervention, 11 of which required hemostasis therapy and rehydration, 2 of which needed blood transfusions, and 1 which required intervention with surgery. Besides, abdominal pain occurred in 108 patients, of which 21 patients had mild pain that relieved after rest, 7 patients had moderate pain that interfered with sleep, and 8 patients had severe pain that required painkillers. Meanwhile, 6 and 2 patients had mild gastrointestinal stress and fever, respectively. However, only one patients presented with mild pain in TJLBs.

**Table 1:** Baseline characteristics and complications of patients before and after matching

Characteristic	Before propensity score matching			After propensity score matching		
	PLB (n = 2711)	TJLB (n = 30)	P	PLB (n = 50)	TJLB (n = 25)	P
Age, > 50 years (%)	1144 (42.2)	19 (63.3)	0.020	30 (60.0)	15 (60.0)	1.000
Gender, male (%)	1508 (55.6)	17 (56.7)	0.909	28 (56.0)	14 (56.0)	1.000
Cirrhosis (%)	202 (7.5)	8 (26.7)	<0.001	17 (34.0)	8 (32.0)	0.862
PH (%)	295 (10.9)	29 (96.7)	<0.001	48 (96.0)	24 (96.0)	1.000
TB (%)						
≤ 2×ULN	2176 (80.3)	19 (63.3)	0.019	35 (70.0)	18 (72.0)	0.871
2-10×ULN	459 (16.9)	9 (30.0)		11 (22.0)	5 (20.0)	
> 10×ULN	76 (2.8)	2 (6.7)		4 (8.0)	2 (8.0)	
Alb (%)						
≤ 30 g/l	44 (1.6)	2 (6.7)	0.032	4 (8.0)	2 (8.0)	1.000
> 30 g/l	2667 (98.4)	28 (93.3)		46 (92.0)	23 (92.0)	
ALT (%)						
≤ 2×ULN	1622 (59.8)	26 (86.7)	0.003	48 (96.0)	23 (92.0)	0.470
2-5×ULN	692 (25.5)	3 (10.0)		2 (4.0)	2 (8.0)	
> 5×ULN	397 (14.6)	1 (3.3)		0 (0)	0 (0)	
AST (%)						
≤ 2×ULN	1983 (73.1)	24 (80.0)	0.350	45 (90.0)	22 (88.0)	0.793
2-5×ULN	494 (18.2)	5 (16.7)		5 (10.0)	3 (12.0)	
> 5×ULN	234 (8.6)	1 (3.3)		0 (0)	0 (0)	
γ-GT (%)						
≤ 2×ULN	1340 (49.4)	15 (50.0)	0.378	32 (64.0)	14 (56.0)	0.434
2-5×ULN	753 (27.8)	13 (43.3)		18 (36.0)	10 (40.0)	
> 5×ULN	518 (22.8)	2 (6.7)		0 (0)	1 (4.0)	
ALP (%)						
≤ 2×ULN	2294 (84.6)	22 (73.3)	0.092	41 (82.0)	19 (76.0)	0.543
2-5×ULN	358 (13.2)	7 (23.3)		9 (18.0)	6 (24.0)	
> 5×ULN	59 (2.2)	1 (3.3)		0 (0)	0 (0)	
Creatinine (%)						
≤ ULN	2660 (98.1)	29 (96.7)	0.567	50 (100.0)	25 (100.0)	−
1-1.5×ULN	35 (1.3)	1 (3.3)		0 (0)	0 (0)	
> 1.5×ULN	16 (0.6)	0 (0)		0 (0)	0 (0)	
Anti-coagulation (%)	14 (0.5)	1 (3.3)	0.403	0 (0)	0 (0)	−
Anti-platelet (%)	36 (1.3%)	0 (0)	1.000	0 (0)	0 (0)	−
Hemodialysis (%)	14 (0.5)	0 (0)	1.000	0 (0)	0 (0)	−
Number of passes (%)						
1	660 (24.3)	2 (6.7)	<0.001	4 (8.0)	2 (8.0)	1.000
2	1976 (72.9)	15 (50.0)		30 (60.0)	15 (60.0)	
≥ 3	75 (2.8)	13 (43.3)		16 (32.0)	8 (32.0)	
Comorbidities (%)	630 (23.2)	8 (26.7)	0.659	10 (20.0)	6 (24.0)	0.690
Hypertension	445 (16.4)	2 (6.7)	0.235	7 (14.0)	2 (8.0)	0.706
Diabetes	238 (8.8)	5 (16.7)	0.235	8 (16.0)	3 (12.0)	0.908
Chronic kidney disease	34 (1.3)	0 (0)	1.000	1 (2.0)	0 (0)	1.000
Cerebrovascular disease	29 (1.1)	0 (0)	1.000	0 (0)	0 (0)	−
Coronary heart disease	63 (2.3)	1 (3.3)	0.510	0 (0)	1 (4.0)	0.333
RBC (×10^12^/L)	4.4 (3.9, 5.2)	3.8 (3.4, 4.6)	0.002	3.9 ± 0.7	3.9 ± 0.9	0.694
Hb (g/L)	132.0 (119.0, 147.0)	113.0 (101.0, 138.3)	0.001	119.9 ± 22.3	114.0 ± 26.2	0.315
PLT (×10^9^/L)	171.0 (127.0, 223.0)	68.0 (40.0, 96.5)	<0.001	101.5 (75.0, 209.5)	64.0 (38.0, 81.5)	<0.001
PT (s)	11.8 (11.0, 12.6)	13.7 (13.1, 14.8)	<0.001	12.2 (11.3, 13.2)	13.7 (13.2, 14.9)	<0.001
INR	1.04 (0.98, 1.11)	1.20 (1.14, 1.29)	<0.001	1.10 (1.00, 1.27)	1.21 (1.16, 1.31)	<0.001
FIB (g/L)	2.3 (1.9, 2.7)	1.9 (1.6, 2.6)	0.014	2.2 (1.7, 2,8)	1.8 (1.5, 2.4)	0.074
Ascites (%)	124 (4.6)	22 (73.3)	<0.001	19 (38.0)	18 (72.0)	0.005
Complication (%)	151 (5.6)	1 (3.3)	0.896	5 (10.0)	1 (4.0)	0.652
Hepatic hemorrhage	35 (1.3)	0 (0)	1.000	2 (4.0)	0 (0)	0.550
Grade 1	21 (0.8)	0 (0)	−	1 (2.0)	0 (0)	−
Grade 2	11 (0.4)	0 (0)		1 (2.0)	0 (0)	
Grade 3	2 (0.1)	0 (0)		0 (0)	0 (0)	
Grade 4	1 (0.0)	0 (0)		0 (0)	0 (0)	
Abdominal pain	108 (4.0)	1 (3.3)	1.000	3 (6.0)	1 (4.0)	1.000
Grade 1	93 (3.4)	1 (3.3)	0.690	3 (6.0)	1 (4.0)	−
Grade 2	7 (0.3)	0 (0)		0 (0)	0 (0)	
Grade 3	8 (0.3)	0 (0)		0 (0)	0 (0)	
Gastrointestinal stress	6 (0.2)	0 (0)	1.000	0 (0)	0 (0)	−
Fever	2 (0.1)	0 (0)	1.000	0 (0)	0 (0)	−

Data are reported as mean ± SD, median (Q1,Q3) or number (percentage)

PLB, percutaneous liver biopsy; TJLB, transjugular liver biopsy; PH, portal hypertension; TB, total bilirubin; Alb, albumin; ALT, alanine aminotransferase; AST, aspartate aminotransferase; γ-GT, γ-glutamine transpeptidase; ALP, alkaline phosphatase; RBC, red blood cell; Hb, hemoglobin; PLT, platelet; PT, prothrombin time; INR, international normalized ratio; FIB, fibrinogen

### Clinical characteristics of patients after propensity score matching

After propensity score matching, 25 pairs of patients were successfully matched. There was no statistical difference in matching factors (P>0.05), as shown in Table 1. Five TJLBs failed to match.

### Complications after propensity score matching

After matching, one (4.0%) and five (10.0%) patients had complications in TJLBs and PLBs, respectively (P > 0.05) (Table 1). The only patient with TJLB presented with mild abdominal pain in the local area and no postoperative bleeding, although TJLB patients had lower platelet counts and poorer coagulation parameters (P < 0.05). However, of the five PLBs, three patients suffered mild pain and two patients had bleeding. One of the bleeding cases required hemostasis therapy and rehydration (Grade 2), while the other needed no intervention (Grade1).

### Specimen quality and diagnostic quality

**Fig. 2:**
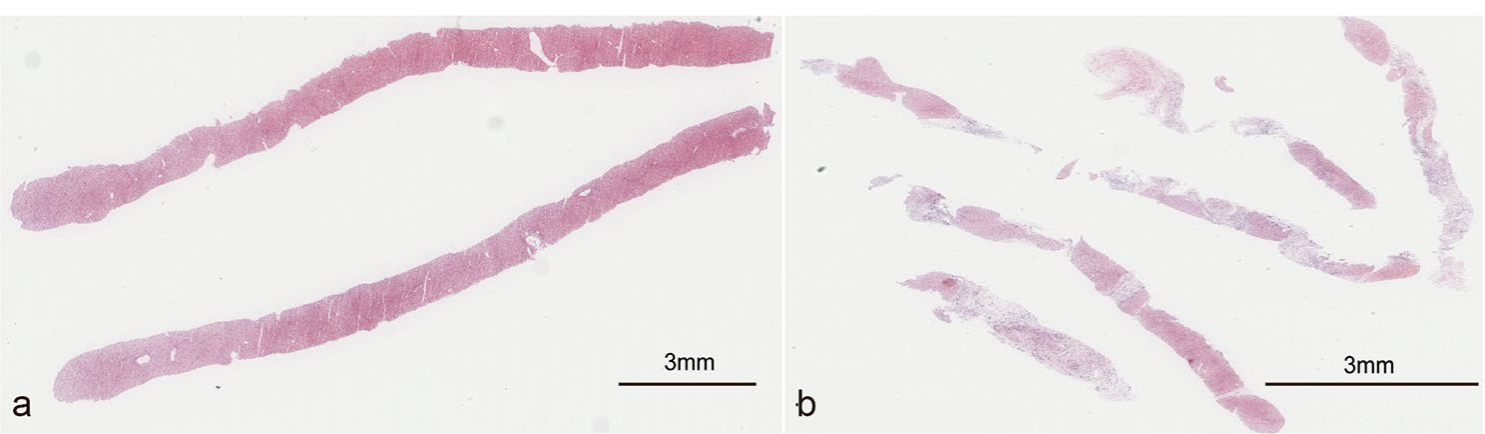
Liver specimens of biopsy. (**a**) The two liver specimens of percutaneous liver biopsy are 20 mm in length and contain a total of 13 portal tracts. HE stained, scale bar, 3 mm. (**b**) The five liver specimens of transjugular liver biopsy are 4-10 mm in length with a total of 10 portal tracts. HE stained, scale bar, 3 mm

Table 2 displayed the liver sample quality of the study population. Overall, patients with PLB had higher-quality specimens than those with TJLB (P < 0.05). Before PSM, the median of CPTs and length of specimens were 6.0 and 10.0 mm in TJLBs, and 10.0 and 15.0 mm in PLBs (P<0.05). After PSM, the median of CPTs between the two groups were 6.0 (TJLB) versus 10.0 (PLB). And the median length of sample in TJLB and PLB patients were 10.0 and 16.5 mm (P<0.05). Compared with PLB, TJLB had more number of passes (P<0.05). Figure 2 showed the liver specimens from PLB and TJLB, separately.

**Table 2:** Liver specimen quality of patients with PLB and TJLB

Pathological characteristics	Before propensity score matching			After propensity score matching		
	PLB (n = 2711)	TJLB (n = 30)	P	PLB (n = 50)	TJLB (n = 25)	P
CPTs	10.0 (8.0,12.0)	6.0 (5.0,8.0)	< 0.001	10.0 (8.0,12.3)	6.0 (5.0,8.0)	< 0.001
Length of sample	15.0 (12.0,18.0)	10.0 (8.0,12.0)	< 0.001	16.5 (14.0,20.0)	10.0 (10.0,12.0)	< 0.001

Data are reported as either mean ± SD or median (Q1,Q3).

PLB, percutaneous liver biopsy; TJLB, transjugular liver biopsy; CPTs, complete portal tracts

At our center, the purpose of liver biopsy in 589 patients (TJLB vs. PLB = 2 vs. 587) was to clarify the staging and grading of viral hepatitis, and in 2152 patients (TJLB vs. PLB = 28 vs. 2124) to investigate the cause of hepatopathy of unknown etiology.

Before PSM, the patients with definite diagnoses in TJLB and PLB group were 27 (96.4%) and 1999 (94.1%), respectively (Table 3). After PSM, the diagnosis rate of hepatopathy of unknown etiology of TJLB and PLB were 95.7% and 93.2% (P>0.05).

**Table 3:** Postoperative diagnosis analysis

Diagnosis	Before propensity score matching					After propensity score matching			
	N (PLB/TJLB)	PLB	TJLB	P		N (PLB/TJLB)	PLB	TJLB	P
Etiology (%)	2711/30					50/25			
Viral hepatitis		587 (21.7)	2 (6.7)	< 0.001			6 (12.0)	2 (8.0)	0.777
Autoimmune liver disease		849 (31.3)	10 (33.3)				16 (34.0)	9 (36.0)	
ALD		60 (2.2)	2 (6.7)				3 (6.0)	2 (8.0)	
Vascular liver disease		144 (5.3)	15 (50.0)				16 (32.0)	11 (44.0)	
NAFLD		441 (16.3)	0 (0)				4 (8.0)	0 (0)	
DILI		440 (16.2)	0 (0)				2 (4.0)	0 (0)	
IMLD		65 (2.4)	0 (0)				0 (0)	0 (0)	
Unknown		125 (4.6)	1 (3.3)				3 (6.0)	1 (4.0)	
Diagnostic quality^a^ (%)	2124/28					44/23			
success		1999 (94.1)	27 (96.4)	0.910			41 (93.2)	23 (95.7)	1.000
failed		125 (5.9)	1 (3.6)				3 (6.8)	1 (4.3)	

Data are reported as number (percentage)

ALD, alcoholic liver disease; NAFLD, non-alcoholic fatty liver disease; DILI, drug-induced liver injury; IMLD, inherited metabolic liver disease

^a^ Diagnostic quality means that that the proportion of patients with a definite cause after liver biopsy, excluding those who had liver biopsy for definite viral hepatitis staging

## Discussion

As an invasive diagnostic technique, the complications and diagnostic efficiency of TJLB were primary concerns for clinical practitioners. The minor complication rate was 6.5%, including neck hematoma, mis-puncture of the carotid artery, small intrahepatic hematoma, and hepatic pain. Major complications occurred in 0.5% of patients, including ventricular arrhythmia, severe intra-abdominal bleeding, and peripheral blood vessel and organ injury [2].

In our center, the rate of complication (3.3%) was lower than previous studies in TJLBs [2]. Small sample sizes might explain the difference. In PLBs, 152 (5.6%) patients were found to suffer complications; 108 (4.0%) presented with abdominal pain, 35 showed with (1.3%) hepatic hemorrhage, 8 (0.3%) suffered mild fever or gastrointestinal stress. After matching, the rate was 4.0% versus 10.0% (TJLB vs. PLB, P>0.05). Regardless of the matching before and after, there was no statistically significant difference in complication rates. Of note, PLB still had a higher rate of complications than TJLB, particularly bleeding events and abdominal pain. It is clinically significant. Also, despite lower platelet counts and higher INR after matching (P<0.05), there was still no bleeding events in TJLBs compared to PLBs.

The success rate of TJLB technique was 100% in this study. However, some cases were reported in the literature of failure of liver biopsy due to stenosis of the inferior vena cava of the hepatic segment or a slight angle between the hepatic vein and the inferior vena cava [9, 10]. These underlined the importance of preoperative radiography, which identified the puncture site and direction according to the specific anatomy of the patient’s liver and improved the success of the puncture while avoiding damage to surrounding vessels and organs. Due to the different access routes, patients may develop severe arrhythmias intraoperatively or postoperatively (via the right atrium). Therefore, compared to PLB, TJLB requires an extremely high level of operator skill.

The quality of the sample is vital to the “gold standard” status of liver biopsy. Good liver tissue should be at least 20 mm in length and contain more than 10 CPTs [4]. But a tissue length of ≥ 15 mm and 6 CPTs are sufficient for diagnosis [11]. Although sample quality of TJLB was not as good as that of PLB after matching (P < 0.001), it can meet the diagnostic requirement of most patients (95.7%). Yet, the sample quality of TJLB remained to be improved. However, Sue MJ et al. showed that the specimens quality obtained by the two procedures was comparable [12]. This may be related to the individual’s condition. Patients had worse baseline conditions in TJLBs compared with PLBs at our center. Cirrhosis and portal hypertension even accounted for 73.3% and 96.7% (P<0.05), respectively. These may be manifested histologically by pseudolobule formation, abnormal distribution, and widening of the intervals of the portal tracts, thus decreasing the number of CPTs [13]. In addition, patients with cirrhosis may be easier to fragment the sample due to pulling during puncture as the fibrous tissue is more brittle [14]. Another critical cause may be the thinner needle (18G) used for TJLB. The average distance between the portal tracts of liver tissue and the central vein is approximately 0.8 mm, and a needle of 0.8 to 1.0 mm in diameter will likely be required to obtain complete portal tracts, of which the use of a 16G needle (PLB) makes this possible [5]. These may explain the number of CTPs in TJLBs was fewer than that in PLBs (P < 0.001).

Inadequate portal tracts will underestimate the grade of fibrosis and even inflammation. Colloredo et al. showed that the diagnosis rate of bridging fibrosis was lowered by half when needle diameter reduced from 1.4 mm to 1.0 mm, or sample length reduced from 30 mm to 10 mm [15]. Furthermore, with the prevalence of non-alcoholic steatohepatitis (NASH), the number of portal tracts is critical. Compared with simple hepatic steatosis, steatohepatitis has a more rapid progression to cirrhosis [16]. And early diagnosis of significant fibrosis and advanced fibrosis can improve prognosis. Moreover, autoimmune hepatitis (AIH) complicated with steatohepatitis presents with moderate to severe interfacial hepatitis or even severe lobular inflammation. Thus, PLB plays an important role in determining severity of fibrosis and inflammation.

In this study, most patients (92.0%) had two or more passes for the biopsy, which can provide more CPTs and extra length to improve diagnosis. Meanwhile, 88.0% of patients with cirrhosis performed multiple passes after matching (≥ 2).

In summary, PLB is more applicable to clarify diagnosis and determine severity of fibrosis and inflammation, whereas TJLB is widely used in patients with severe coagulation disorders and massive ascites. Although TJLB might have fewer CPTs compared with PLB, multiple passes can be an appropriate choice.

Regarding diagnostic yield, whether matched or not, the two procedures had comparable diagnostic quality. Similar to the previously reported studies, the diagnosis rate of hepatopathy of unknown etiology of TJLB versus PLB before and after matching were 96.4% vs. 94.1% and 95.7% vs. 93.2%, respectively (P > 0.05) [12, 17].

## Conclusion

Transjugular liver biopsy is a safe and effective invasive diagnostic technique that expands indications of liver biopsy with reliable diagnostic quality when patients had equal levels of liver function and number of passes compared with percutaneous liver biopsy.

## Data Availability

The datasets used and analyzed during the current study are available from the corresponding author on request.

## Abbreviations

LB: Liver biopsy
PLB: Percutaneous liver biopsy
TJLB: Transjugular liver biopsy
INR: International normalized ratio
PTA: Prothrombin time activity
HVPG: Hepatic venous pressure gradients
PSM: Propensity score matching
PLT: Platelet
PH: Portal hypertension
CPTs: Complete portal tracts
TB: Total bilirubin
Alb: Albumin
ALT: Alanine aminotransferase
AST: Aspartate aminotransferase
ALP: Alkaline phosphatase
γ-GT: γ-glutamine transpeptidase
PT: Prothrombin time
IJV: Internal jugular vein
ALD: Alcoholic liver disease
NAFLD: Non-alcoholic fatty liver disease
DILI: Drug-induced liver injury
IMLD: Inherited metabolic liver disease
NASH: Non-alcoholic steatohepatitis
AIH: Autoimmune hepatitis

## Declarations

**Consent of publication**

## References

[1] Bissonnette J, Riescher-Tuczkiewicz A, Gigante E, Bourdin C, Boudaoud L, Soliman H, Durand F, Ronot M, Valla D, Vilgrain V (2022). Predicting bleeding after liver biopsy using comprehensive clinical and laboratory investigations: a prospective analysis of 302 procedures. J Thromb Haemost.

[2] Chi H, Hansen BE, Tang WY, Schouten JN, Sprengers D, Taimr P, Janssen HL, de Knegt RJ (2017). Multiple biopsy passes and the risk of complications of percutaneous liver biopsy. Eur J Gastroenterol Hepatol.

[3] Khalifa A, Rockey DC (2020). The utility of liver biopsy in 2020. Curr Opin Gastroenterol.

[4] Neuberger J, Patel J, Caldwell H, Davies S, Hebditch V, Hollywood C, Hubscher S, Karkhanis S, Lester W, Roslund N (2020). Guidelines on the use of liver biopsy in clinical practice from the british Society of Gastroenterology, the Royal College of Radiologists and the Royal College of Pathology. Gut.

[5] Rockey DC, Caldwell SH, Goodman ZD, Nelson RC, Smith AD (2009). American Association for the study of liver D: liver biopsy. Hepatology.

[6] De Gottardi A, Rautou PE, Schouten J, Rubbia-Brandt L, Leebeek F, Trebicka J, Murad SD, Vilgrain V, Hernandez-Gea V, Nery F (2019). Porto-sinusoidal vascular disease: proposal and description of a novel entity. Lancet Gastroenterol Hepatol.

[7] de Franchis R, Bosch J, Garcia-Tsao G, Reiberger T, Ripoll C, Baveno VIIF (2022). Baveno VII - renewing consensus in portal hypertension. J Hepatol.

[8] Terjung B, Lemnitzer I, Dumoulin FL, Effenberger W, Brackmann HH, Sauerbruch T, Spengler U (2003). Bleeding complications after percutaneous liver biopsy. An analysis of risk factors. Digestion.

[9] Dohan A, Guerrache Y, Dautry R, Boudiaf M, Ledref O, Sirol M, Soyer P (2015). Major complications due to transjugular liver biopsy: incidence, management and outcome. Diagn Interv Imaging.

[10] Kalambokis G, Manousou P, Vibhakorn S, Marelli L, Cholongitas E, Senzolo M, Patch D, Burroughs AK (2007). Transjugular liver biopsy–indications, adequacy, quality of specimens, and complications–a systematic review. J Hepatol.

[11] Ble M, Procopet B, Miquel R, Hernandez-Gea V, Garcia-Pagan JC (2014). Transjugular liver biopsy. Clin Liver Dis.

[12] Sue MJ, Lee EW, Saab S, McWilliams JP, Durazo F, El-Kabany M, Kaldas F, Busuttil RW, Kee ST (2019). Transjugular Liver Biopsy: safe even in patients with severe Coagulopathies and multiple biopsies. Clin Transl Gastroenterol.

[13] Stift J, Semmler G, Walzel C, Mandorfer M, Schwarzer R, Schwabl P, Paternostro R, Scheiner B, Woran K, Pinter M (2019). Transjugular aspiration liver biopsy performed by hepatologists trained in HVPG measurements is safe and provides important diagnostic information. Dig Liver Dis.

[14] Behrens G, Ferral H (2012). Transjugular liver biopsy. Semin Intervent Radiol.

[15] Coral GP, Antunes AD, Serafini AP, Araujo FB, Mattos AA (2016). Liver biopsy: importance of Specimen size in the diagnosis and staging of chronic viral Hepatitis. Rev Inst Med Trop Sao Paulo.

[16] Singh S, Allen AM, Wang Z, Prokop LJ, Murad MH, Loomba R (2015). Fibrosis progression in nonalcoholic fatty liver vs nonalcoholic steatohepatitis: a systematic review and meta-analysis of paired-biopsy studies. Clin Gastroenterol Hepatol.

[17] Donmez H, Kahriman G, Ozcan N, Mavili E, Deniz K (2012). Transjugular liver biopsy: results of 97 patients. Balkan Med J.

